# Metagenomic Evaluation of Oral Microbiota in Patients Affected by Oral Lichen Planus: A Pilot Study

**DOI:** 10.1111/odi.70296

**Published:** 2026-03-17

**Authors:** A. Dormiente, L. Mancabelli, M. Ventura, G. Longhi, G. Mergoni, M. Manfredi

**Affiliations:** ^1^ Dentistry Unit, Department of Medicine and Surgery University Hospital of Parma, University of Parma Parma Italy; ^2^ Laboratory of Probiogenomics, Department of Chemistry, Life Sciences and Environmental Sustainability University of Parma Parma Italy

**Keywords:** corticosteroids, dysbiosis, metagenomics, oral lichen planus, oral microbiota

## Abstract

**Objective:**

To characterize the oral bacterial and fungal microbiota of symptomatic oral lichen planus patients undergoing topical corticosteroid therapy and to explore microbial patterns potentially associated with subsequent oral candidiasis.

**Methods:**

Twelve patients with clinically and histologically confirmed OLP were enrolled. Unstimulated saliva and tongue dorsum swabs were collected at baseline and weekly for three weeks during betamethasone therapy. Bacterial communities were profiled using shallow shotgun metagenomics analyzed with METAnnotatorX2, and fungal communities through ITS sequencing and QIIME 2 workflows. Beta‐diversity analyses assessed the effects of time, clinical variables, and later candidiasis development. Associations between bacterial taxa and *Candida* abundance were evaluated using Spearman correlations.

**Results:**

Oral candidiasis developed in three patients (25%). Microbiota composition showed marked inter‐individual variability but high intra‐subject stability, with no consistent shifts linked to corticosteroid therapy. Commensal taxa such as *Streptococcus*, *Rothia*, and *Actinomyces* were negatively associated with candidiasis and Candida abundance, whereas anaerobic species including *Porphyromonas gingivalis* , *Prevotella multiformis* , and *Lachnoanaerobaculum gingivalis* displayed positive correlations with fungal proliferation.

**Conclusions:**

Despite overall stability of the oral microbiota during therapy, specific bacterial signatures were associated with subsequent *Candida* overgrowth. These findings suggest that cross‐kingdom interactions may influence susceptibility to corticosteroid‐associated candidiasis and warrant validation in larger cohorts.

## 1 Introduction

Oral lichen planus (OLP) is a chronic inflammatory disorder of the oral mucosa, generally considered to be of autoimmune origin. It affects approximately 1%–2% of the general population (González‐Moles et al. 2021), with a clear female predominance and peak incidence between the fourth and sixth decades of life (Andreasen 1968). Clinically, OLP manifests as bilateral and often symmetrical lesions that can involve various oral sites, most frequently the buccal mucosa, tongue, and gingiva (Mignogna et al. 2005). The clinical spectrum of OLP includes asymptomatic reticular forms as well as symptomatic atrophic and erosive variants, the latter being associated with significant pain, burning sensations, and impaired oral function and quality of life (Eisen et al. 2005).

The aetiology of OLP remains incompletely understood. However, it is widely accepted that the disease is mediated by a T cell‐driven immune response targeting basal keratinocytes (El‐Howati et al. 2023). Various contributing factors have been proposed, including genetic predisposition (Carrozzo et al. 2004), psychological stress (Na et al. 2016), systemic diseases (Ivanovski et al. 2005), and chronic infections such as hepatitis C virus (Pilli et al. 2002). Moreover, the Koebner phenomenon, lesions developing in response to trauma, may explain the frequent localization of OLP in mechanically stressed areas like the tongue and buccal mucosa (Thongprasom and Dhanuthai 2008). Histopathological confirmation is often required to establish a definitive diagnosis and to exclude other vesiculo‐erosive conditions or dysplastic changes (Al‐Hashimi et al. 2007).

Importantly, OLP is recognized as a potentially malignant disorder by the World Health Organization, with an estimated risk of malignant transformation ranging between 0.44% and 2.28% (Warnakulasuriya et al. 2007). This risk is higher in the erosive subtype and in lesions involving the tongue (González‐Moles et al. 2020). Consequently, long‐term clinical follow‐up is warranted in all patients.

Topical corticosteroids are considered the gold standard for the management of symptomatic OLP due to their anti‐inflammatory and immunosuppressive properties (Lodi et al. 2020). Despite their clinical efficacy, prolonged corticosteroid use is not without adverse effects. One of the most frequent complications is the development of oral candidiasis, which has been reported in 13%–25% of patients undergoing corticosteroid treatment (Jainkittivong et al. 2007; Alqahtani and Alabeedi 2023). The local immunosuppressive action of these drugs, particularly on cell‐mediated immunity, facilitates fungal overgrowth and contributes to worsening symptoms and therapeutic resistance (Marable et al. 2016).

In recent years, the role of the oral microbiota, referring to the bacterial component of the oral cavity, in health and disease has gained increasing attention. However, the oral cavity hosts a highly diverse and dynamic microbial ecosystem, encompassing not only bacteria, but also fungi, viruses, and archaea (Ghannoum et al. 2010). This microbial community plays a crucial role in maintaining mucosal homeostasis, regulating immune responses, and preventing colonization by pathogens (Caselli et al. 2020). Disruptions of this balance, referred to as dysbiosis, have been implicated in various oral and systemic diseases, including periodontitis, candidiasis, and potentially OLP (Darveau 2010; Kurago 2016).

With the advent of next‐generation DNA sequencing technologies and metagenomic analyses, our understanding of the complexity of the oral microbiota has significantly improved. Several studies have demonstrated alterations in the oral bacterial and fungal communities in patients with OLP compared to healthy controls (Wang et al. 2020; Li et al. 2019), suggesting a potential link between microbial dysbiosis and disease pathogenesis. Some evidence also points to a correlation between microbial composition and disease severity or subtype, although findings remain inconsistent and limited by cross‐sectional study designs (Jung and Jang 2022).

Even fewer studies have investigated how therapeutic interventions, particularly topical corticosteroid therapy, affect the structure and function of the oral microbiota over time. This gap of knowledge is particularly relevant considering the risk of secondary infections, such as candidiasis, which may be influenced by pre‐existing microbial profiles or treatment‐induced dysbiosis (Kragelund and Keller 2019; Marlina et al. 2022).

A recent systematic review and meta‐analysis confirmed a significant association between *Candida albicans* colonization and OLP (OR = 4.16, 95% CI: 1.28–13.51, *p* = 0.024), and reported a near‐significant association with broader oral microbiota dysbiosis (OR = 11.74, 95% CI: 0.65–210.71, *p* = 0.059), particularly in erosive forms of OLP (Israyani et al. 2025). These findings highlight the complex microbial dynamics involved in the disease and underscore the need for longitudinal studies assessing microbiota changes during treatment.

This preliminary observational study aims to explore longitudinal changes in the oral microbiota, encompassing both bacterial and communities of *Candida* spp., in symptomatic OLP patients treated with topical corticosteroids. By analyzing samples collected at multiple time points during therapy, this investigation aims to generate early insights into how corticosteroid treatment may influence oral microbial dynamics in each patient, with particular attention to potential dysbiosis and secondary infections such as candidiasis. The findings, even if preliminary, may contribute to future, larger‐scale studies investigating microbiota‐informed approaches to OLP management.

## 2 Materials and Methods

The study was approved by the AVEN (Area Vasta Emilia Nord) Ethical Committee of the Dentistry Units at the University Hospital of Parma (study number: 703/2021/TESS/AOUPR). The selection of OLP patients enrolled in the study was carried out among those normally followed at the Dentistry Unit of the University Hospital of Parma. Although the study involves a second site (San Paolo ASST Hospital, Milan), patient enrollment at that center is still ongoing, and the present data refer exclusively to the Parma Unit.

### 2.1 Inclusion and Exclusion Criteria

Patients included in the study met the following inclusion criteria: adult subjects (> 18 years old), consecutively evaluated and presenting with symptomatic OLP requiring topical corticosteroid therapy.

Exclusion criteria were:

– Non‐symptomatic OLP
– Current or recent use of topical and/or systemic corticosteroid therapy
– Use of corticosteroids, antifungals, antibiotics, or systemic/topical immunosuppressants within the past three months
– Pregnancy or lactation

The diagnosis of OLP had been previously confirmed by both clinical and histopathological criteria. Eligible patients were informed about the study and, upon agreeing to participate, provided written informed consent. Patients who declined participation were managed according to standard care.

### 2.2 Sample Size

The sample size was calculated based on data from Lodi et al. (2007), assuming a 50% incidence of candidiasis at three weeks. To detect an incidence of at least 20% (power 80%, alpha 0.05), 21 patients were required. A total of 24 patients were planned for enrollment across both clinical centers (12 in Parma and 12 in Milan). At the time of this analysis, only the 12 patients enrolled at the Parma site had completed the study protocol, and their data are presented here as preliminary results.

### 2.3 Data and Sample Collection

For each patient, the following data were collected: sex, age, relevant medical history, smoking habits, clinical and histological diagnosis of OLP, and symptomatology (recorded as YES/NO, categorical, and using a 0–10 VAS; Visual Analogue Scale, 0–10 cm, with 0 = no pain and 10 = extreme pain). Oral hygiene status was assessed using the Plaque Control Record (O'Leary et al. 1972), evaluating plaque presence on the four surfaces of each tooth (buccal, lingual, mesial, distal). The plaque index was calculated as the percentage of affected surfaces.

Periodontal status was assessed at baseline by clinical periodontal examination (probing pocket depth, clinical attachment level, bleeding on probing) and radiographic evaluation when available, and classified according to the 2017 World Workshop on the Classification of Periodontal and Peri‐Implant Diseases and Conditions.

Sample collection was performed during routine visits scheduled for OLP patients. In the morning, at least 2 h after oral hygiene and 1 h after eating or drinking, each patient provided an oral sample consisting of at least 2 mL of unstimulated whole saliva, collected by passive drooling, and a tongue dorsum swab. Patients were instructed not to use mouthwash or products other than toothpaste prior to the visit.

Following initial sample collection (T0), topical corticosteroid therapy was prescribed to patients as betamethasone 1 mg effervescent tablets. Patients were instructed to dissolve two 1 mg tablets in 30 mL of water per rinse (2 mg/rinse) and to perform three rinses daily. They were re‐evaluated at one (T1), two (T2), and three weeks (T3) after therapy initiation. Saliva and tongue swab samples were repeated at each of these follow‐up visits. The fourth‐week visit was conducted as a clinical follow‐up without microbiological sampling.

Oral candidiasis was diagnosed clinically during follow‐up based on the presence of characteristic signs (e.g., pseudomembranous white plaques on the lingual dorsum and/or erythematous lesions) in association with compatible symptoms (e.g., burning sensation). In such cases, a final oral sample was collected, and the patient exited the study to receive antifungal treatment according to standard clinical practice.

### 2.4 Sample Handling and Processing

Collected samples were transferred to a sterile vial containing an inactivating liquid to block bacterial replication and inactivate the infectious capacity of viruses. All samples were then transported to the Laboratory of Probiogenomics, Department of Chemistry, Life Sciences and Environmental Sustainability, University of Parma, for further processing.

### 2.5 Taxonomic Classification

The extraction of bacterial DNA was performed using the ZymoBIOMICS DNA Miniprep Kit (Zymo Research), according to the manufacturer's instructions (https://www.qiagen.com/be). The collected samples were then sequenced using NextSeq Illumina shotgun metagenomic methodologies and analyzed to assess microbiota composition at the species level with METAnnotatorX2 software, following the standard filtering parameters reported in the manual, with *Homo sapiens* reads removed (Milani et al. 2021). Afterward, the taxonomic classification of 100,000 reads was achieved using MegaBLAST (Chen et al. 2015), which employed a manually curated and pre‐processed database of genomes retrieved from the National Center for Biotechnology Information, following the METAnnotatorX2 manual (Milani et al. 2021).

### 2.6 *Candida* Identification and Fungal Community Profiling

Fungal DNA was extracted from the same oral samples used for bacterial analysis using the ZymoBIOMICS DNA Miniprep Kit (Zymo Research), following the manufacturer's protocol.

Among the 12 enrolled subjects, sufficient fungal DNA was obtained from 10, while two samples yielded quantities below the detection threshold and were therefore excluded from downstream analysis.

The internal transcribed spacer (ITS) region of the fungal rRNA operon was amplified using primers BITS (5′‐GAGATCCRTTGYTRAAAGTT‐3′) and B58S3 (5′‐ACCTGCGGARGGATCA‐3′) (Agarbati et al. 2019) and sequenced on an Illumina NextSeq. Amplicon data were processed through the QIIME 2 pipeline using default quality filtering parameters (Bokulich et al. 2018). Forward and reverse reads were merged to reconstruct full ITS amplicons, and sequences with a mean quality score < 15 or primer mismatches were discarded. Amplicon sequence variants (ASVs) were defined at 100% sequence homology using DADA2 (Callahan et al. 2016), and low‐abundance ASVs (< 10 reads) were filtered out. Taxonomic assignment was performed using the UNITE database (Kõljalg et al. 2013), and the relative abundance of the *Candida* genus was extracted for downstream correlation analysis with bacterial community profiles.

### 2.7 Statistical Analysis

Statistical analyses were performed using SPSS (version 25) and R (version 4.1.2). Microbial beta‐diversity differences between saliva and tongue swab samples were analyzed using PERMANOVA based on the Bray–Curtis dissimilarity index, implemented in QIIME2 (Kõljalg et al. 2013). ADONIS analysis was performed to assess the effects of specific factors, including sex, age, smoking status, subject identity, biological matrix type, and subsequent candidiasis development on microbial composition.

To investigate potential associations between oral microbiota composition and fungal parameters, Spearman correlation analyses were conducted in R using the Hmisc package. These analyses explored correlations between the development of oral candidiasis and the relative abundance of bacterial taxa, as well as between bacterial abundances and the relative abundance of the *Candida* genus quantified through ITS sequencing. *P*‐values were adjusted for multiple testing using the Benjamini–Hochberg false discovery rate (FDR) correction.

### 2.8 Data Availability

Raw Italian shotgun metagenomic and fungal ITS sequencing data are available through the SRA under the study accession number PRJNA1391436.

## 3 Results and Discussion

### 3.1 Clinical and Demographic Characteristics of the Study Population

A total of 12 patients (10 females and 2 males) were included in this preliminary analysis. The mean age was 65.5 years (±8.4). Two patients (16.7%) were active smokers at the time of inclusion. The most common comorbidities were hypertension (50%), hypothyroidism (25%), and type II diabetes (8%) (Table 1).

**TABLE 1:** Clinical and demographic characteristics of the study population.

Sample	Sex	Age	Smoke	Diseases	Drugs	Type OLP	Symptoms	Sites	Pain	Plaque	Candidiasis	T0	T1	T2	T3
A1	F	78	No	Hypertension, Hypercholesterolemia	Atorvastatin, Bisoprolol, Ramipril, Cardioaspirin	Erosive	Yes	Buccal mucosa, Gingiva	4–6	0%	2a week	+	+	+	
A2	F	76	Yes	Diabetes II, Hypertension	Metformin, Valsartan, Barnidipine	Reticular‐erosive	Yes	Buccal mucosa, Tongue, Gingiva	4–6	0%	2a week	+	+	+	
A3	F	57	No	/	/	Erosive	Yes	Buccal mucosa, Tongue	7–10	0%	3a week	+	+	+	+
A4	F	72	No	Hypothyroidism, Hypertension	Eutirox, Ramipril, Bisoprolol	Reticular‐erosive	Yes	Buccal mucosa, Tongue, Gingiva	4–6	38%	NO	+	+	+	+
A5	M	56	No	Hypothyroidism	Eutirox	Erosive	Yes	Buccal mucosa	4–6	15%	NO	+	+	+	+
A6	F	76	No	Hypertension, Diabetes II, Addison's disease	Eutirox, Cardirene, Insulin	Erosive	Yes	Buccal mucosa	1–3	45%	NO	+	+	+	+
A7	F	72	No	Hypertension, MRGE	Beta‐blocker, Rabeprazol	Erosive	Yes	Buccal mucosa	4–6	40%	NO	+	+	+	+
A8	M	52	Yes	Hypothyroidism	Eutirox	Reticular‐erosive	Yes	Buccal mucosa, Gingiva	4–6	74%	NO	+	+	+	
A9	F	60	No	Hypertension	Bisoprolol	Reticular‐erosive	Yes	Buccal mucosa	1–3	0%	NO	+	+	+	+
A10	M	76	No	Hypertension, Hypercholesterolemia	Triplian, Brilique, Cardicor, Resumibe, Talmusolin	Erosive	Yes	Buccal mucosa, Gingiva	7–10	70%	NO	+	+	+	
A11	F	80	No	Hypertension, Basedow‐Graves disease	Cardirene	Reticular‐erosive	Yes	Buccal mucosa	1–3	20%	NO	+	+	+	+
A12	F	55	No	/	/	Reticular‐erosive	Yes	Buccal mucosa, Tongue	1–3	54%	NO	+	+	+	+

Regarding pharmacological treatment, which is the most frequently reported drug included Ramipril, Eutirox, and Bisoprolol.

Clinically, seven patients (58.3%) presented with erosive OLP, while 5 (41.7%) showed a reticular‐erosive form. The buccal mucosa was affected in all patients (100%), followed by the tongue and gingiva (both 50%). Symptom severity was reported as VAS 4–6 in 75% of cases, and 7–10 in the remaining 25%.

Plaque index values ranged from 0% to 38%, with a median of approximately 8% (Table 1).

Oral candidiasis developed in 3 patients: 2 cases occurred at week 2 (T2) and 1 case at week 3 (T3) of follow‐up. Specifically, two patients developed pseudomembranous candidiasis, characterized by the presence of white plaques on the lingual dorsum, associated with burning symptoms. One patient developed erythematous candidiasis, presenting as red areas on the lingual dorsum and palate, accompanied by intense burning.

As per protocol, patients who developed clinically detectable candidiasis were treated with conventional topical antifungals (nystatin mouth rinses, four times daily for 4 weeks), resulting in the resolution of signs and symptoms in the following weeks.

An overview of the clinical and demographic characteristics is summarized in Table 1.

### 3.2 Oral Microbiota Composition and Its Relationship with Clinical Parameters

The oral microbiota was investigated and characterized using shallow shotgun metagenomic sequencing, performed on DNA extracted from both saliva and lingual swab samples collected at baseline (T0), prior to the initiation of topical corticosteroid therapy. DNA sequencing was performed on the NextSeq Illumina platform, yielding an average of 165,211 ± 138,496 reads per sample (Table S1). After quality filtering, host DNA removal, and taxonomic profiling with METAnnotatorX2, an average of 36,760 ± 33,574 reads per sample were obtained (Table S2), and relative abundances were calculated at the species level (Table S2).

The baseline (T0) profiles revealed complex bacterial communities dominated by taxa typically associated with the oral microbiota, including *Rothia mucilaginosa* (11.2% in saliva and 15.8% in tongue), *Prevotella melaninogenica* (5.4% and 7.3%), and *Porphyromonas pasteri* (4.3% and 6.6%; Table S2). Species belonging to the genera *Schaalia* (4.5% and 5.0%) and *Actinomyces* (4.3% and 3.8%) were also consistently represented (Table S2). Overall, these compositions were in line with microbial signatures commonly reported for the healthy oral cavity (Baker et al. 2024; Mancabelli et al. 2022).

At baseline (T0), beta‐diversity analysis based on Bray‐Curtis distances was performed to evaluate potential intra‐individual differences between saliva and tongue swab samples, as well as the influence of clinical and demographic variables. No systematic compositional separation was observed between the two matrices (PERMANOVA pseudo‐*F* = 0.099, *p*‐value = 0.425; Figure 1a). Consistently, an ADONIS model including several covariates, such as sex, age, smoking status, subject identity, biological matrix type, and future candidiasis development, confirmed the absence of a matrix effect (*R* ^2^ = 0.04, *p*‐value = 0.232) while revealing a significant contribution of the subject itself (*R* ^2^ = 0.18, *p*‐value = 0.001). This indicates that microbial community composition was largely consistent across the two matrices within the same individual, whereas a higher degree of variability was observed between different patients. To further quantify within‐subject variability, Bray‐Curtis dissimilarities were calculated between saliva and tongue samples of the same individual. The resulting distances ranged from 0.17 to 0.46 (average = 0.33; Figure 1a), indicating moderate compositional differences between these closely connected oral niches. In addition, a Spearman correlation analysis including the binary “matrix” variable (saliva = 1; tongue = 0) revealed a significant positive association between saliva and overall species richness (Spearman's rank correlation coefficient = 0.693, *p*‐value < 0.001; FDR‐adjusted *p*‐value < 0.001). This finding indicates that salivary samples harbor a more diverse microbial assemblage than lingual swabs. Biologically, this pattern is consistent with the integrative nature of saliva, which collects microorganisms shed from multiple oral surfaces, thereby providing a broader yet representative snapshot of the oral microbiota (Jo et al. 2019).

**FIGURE 1:**
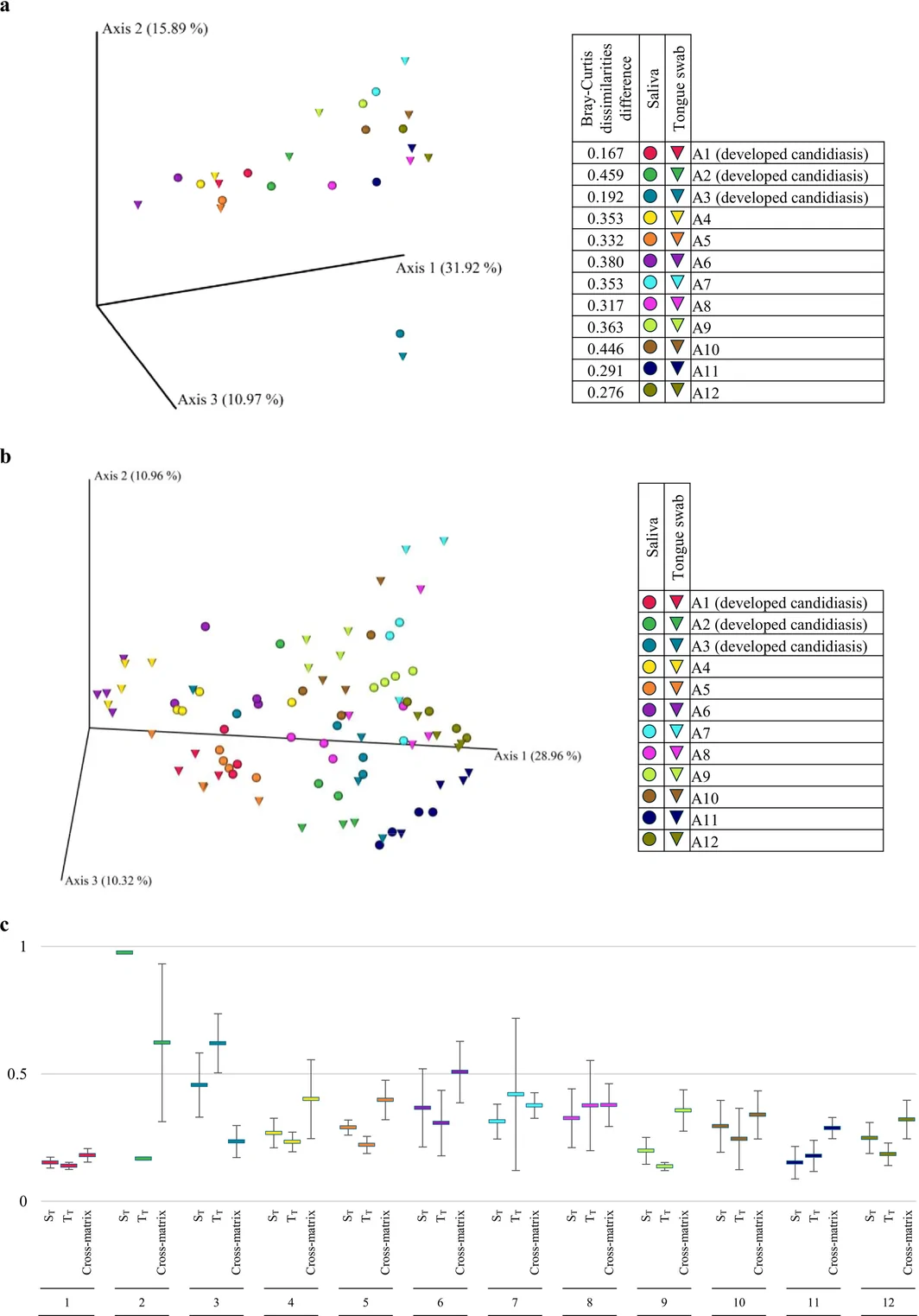
Microbial beta diversity across time points and sample matrices. (a and b) report the Principal Coordinates Analysis (PCoA) based on Bray–Curtis dissimilarity at baseline (T0) and including all available time points (T0–T3), respectively. Figure (c) shows the intra‐patient Bray–Curtis distances calculated across time points and sample matrices, showing overall stability of microbial composition within individuals.

Furthermore, in the ADONIS model, the variable related to future candidiasis development emerged as significantly associated with community structure when all samples at T0 were analyzed jointly, after accounting for sex, age, smoking, subject identity, and matrix effects (*R* ^2^ = 0.12, *p*‐value = 0.003). However, when the analysis was repeated separately for saliva and tongue samples, thus maintaining full independence, the effect did not reach significance (*p*‐value > 0.5), most likely due to the small number of *Candida*‐positive cases and limited statistical power.

Notably, the direction and magnitude of the effect (both *R* ^2^ = 0.09) remained consistent across the independent models, suggesting a reproducible trend that warrants confirmation in larger cohorts.

### 3.3 Temporal Dynamics and Intra‐Subject Stability of the Oral Microbiota

Building on the baseline observations, the analysis was extended to all available time points (T0–T3) to assess longitudinal dynamics of the oral and tongue microbial communities during corticosteroid treatment.

Beta‐diversity analyses based on Bray–Curtis distances confirmed that samples collected from the same individual clustered closely together (Figure 1b), whereas those from different subjects were clearly separated (PERMANOVA pseudo‐*F* = 8.97, *p*‐value = 0.001), indicating that subject‐specific microbial profiles strongly dominated the overall variance. Moreover, pairwise comparisons further supported this finding, with all subjects differing significantly from each other (*q*‐value < 0.05).

Furthermore, specific ADONIS analyses, considering all patients' covariates, revealed no significant effects of time, biological matrix type, or candidiasis development (all *p*‐values > 0.05), suggesting that community structure remained largely stable throughout the study period, with no systematic shifts associated with treatment duration or fungal colonization. Interestingly, only the smoking variable reached nominal significance (*p*‐value = 0.001) but explained only a small fraction of variance (*R* ^2^ = 0.03), and this association was accompanied by unequal dispersion between smokers and non‐smokers (*p*‐value = 0.06), suggesting that the apparent effect may reflect group heterogeneity or limited sample size rather than a true compositional shift.

Intra‐patient Bray–Curtis distances (Figure 1c) indicated that microbial composition remained relatively stable within each individual over time and across matrices. Such stability likely reflects a resilient core oral microbiota maintained throughout treatment, with only subtle and transient perturbations possibly linked to topical corticosteroid exposure or episodic *Candida* overgrowth.

### 3.4 Association Between Oral Microbiota Composition and Candida Development

Given the absence of significant effects of sample type, time, or most clinical covariates on overall community composition, all oral samples collected across time points (T0–T3) were subsequently analyzed together to identify specific bacterial taxa potentially associated with the future development of oral candidiasis. This approach was justified by the high intra‐individual stability observed over time and by the lack of systematic temporal or matrix‐related shifts, indicating that each patient's microbiota could be treated as a consistent compositional unit. A binary variable reflecting whether candidiasis developed during follow‐up was therefore used as the primary outcome. Associations between microbial relative abundances and this variable were explored through non‐parametric Spearman correlation analyses.

The correlation analysis revealed a possible ecological pattern linking bacterial community structure to the subsequent development of oral candidiasis (Figure 2). A broad subset of taxa typically regarded as commensal and health‐associated exhibited significant negative correlations with *Candida* occurrence, indicating a possible negative association with the later development of fungal overgrowth. This group included dominant oxygen‐tolerant genera such as *Streptococcus*, *Rothia*, *Actinomyces*, and *Corynebacterium*, which are known to play key roles in epithelial colonization, maintaining pH balance, and establishing a microaerophilic environment that limits fungal proliferation (Kreth et al. 2024).

**FIGURE 2:**
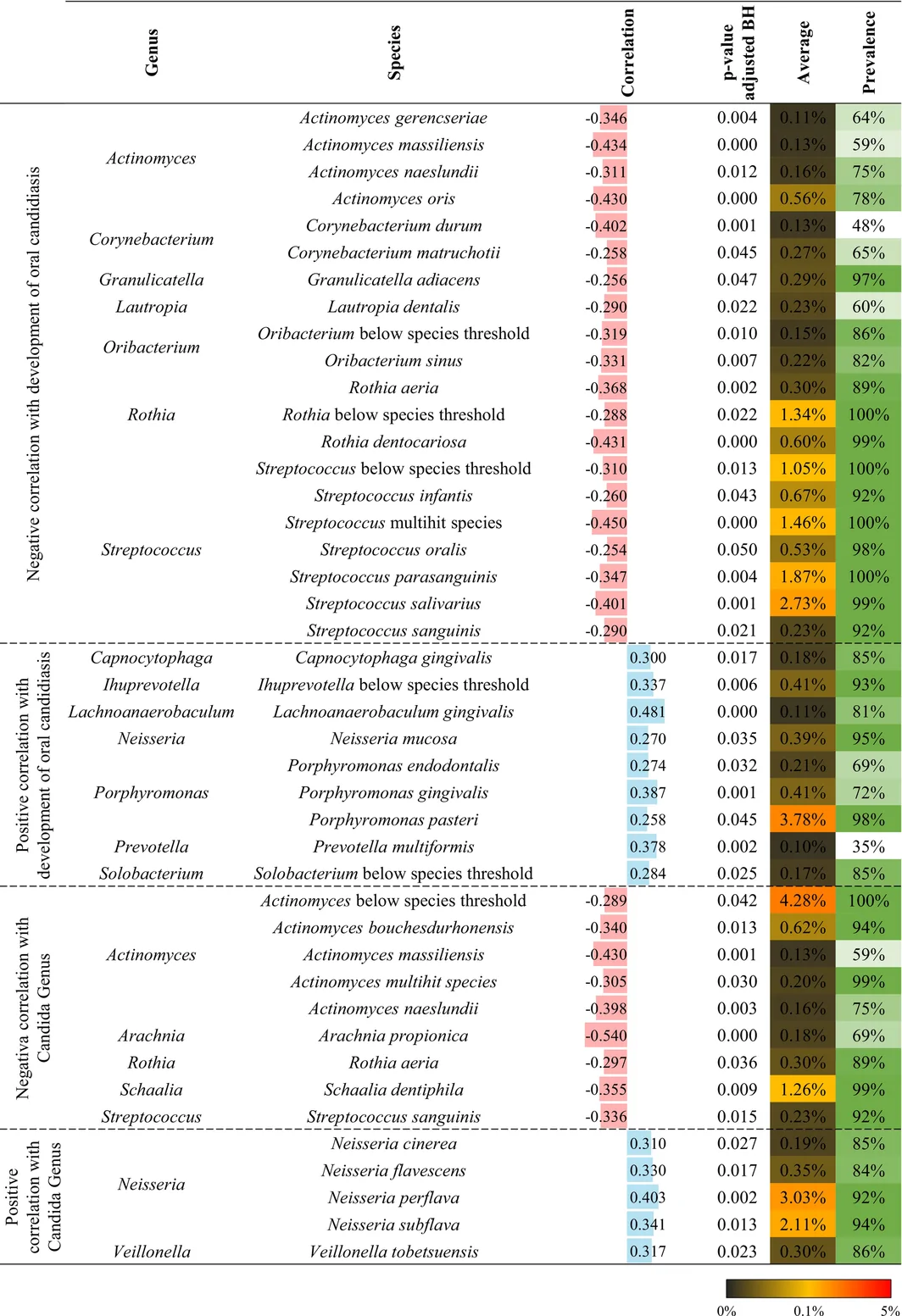
Associations between microbial taxa abundance and *Candida*‐related measures. In detail, the figure reports correlations between selected bacterial taxa, *Candida* abundance, and *Candida*‐related variables. Color intensity represents mean relative abundance and prevalence, while correlation coefficients indicate the strength and direction of the associations.

Conversely, a smaller but distinct cluster of anaerobic and proteolytic bacteria was significantly positively correlated with *Candida* presence, including *Porphyromonas gingivalis* , *Prevotella multiformis* , *Capnocytophaga gingivalis* , and *Lachnoanaerobaculum gingivalis* (Figure 2). These species are characteristic of low‐oxygen, nutrient‐rich environments and are frequently implicated in mucosal dysbiosis and co‐aggregation with *Candida albicans* (Bartnicka et al. 2019).

To further validate these observations, the relative abundance of the *Candida* genus, as derived from ITS sequencing, was correlated with bacterial community profiles (Figure 2 and Table S3). Although fungal DNA could be successfully recovered from only ten individuals, the resulting profiles were consistent and biologically informative. Despite the low biomass and limited resolution of fungal data, *Candida* abundance displayed a highly significant negative correlation with commensal *Actinomyces*, *Rothia*, and *Streptococcus* species, as well as significant positive correlations with facultative and anaerobic genera, such as *Neisseria* and *Veillonella*. The recurrence of these associations across independent *Candida* measures reinforces the biological plausibility of a cross‐kingdom imbalance preceding fungal proliferation (Sedghi et al. 2021).

Although the limited cohort size precluded formal statistical confirmation after multiple‐testing correction, the directional coherence of these correlations supports a biologically plausible model in which the onset of oral candidiasis reflects the culmination of gradual microbial imbalance rather than a discrete infectious event.

Overall, these findings highlight the existence of a dynamic interplay between bacterial and fungal components of the oral microbiota in patients with OLP, suggesting that subtle bacterial community fluctuations may precede or accompany fungal proliferation during treatment.

## 4 Conclusions

This preliminary study provides new insights into the short‐term dynamics of the oral microbiota in patients with symptomatic oral lichen planus (OLP) undergoing topical corticosteroid therapy. Through a combined analysis of bacterial and fungal communities, our results contribute to understanding how host–microbiota interactions evolve under local immunosuppressive conditions and how these changes may predispose to opportunistic infections such as oral candidiasis.

At the global level, microbial composition appeared stable over time and across sampling sites (saliva and tongue dorsum), showing high inter‐individual variability but remarkable intra‐subject consistency. These findings indicate that the oral microbiota of OLP patients, although potentially altered by chronic mucosal inflammation, maintains a relatively stable structure during corticosteroid therapy. We did not identify a single, distinct baseline (T0) microbiota profile that predisposed individuals to oral candidiasis. However, the cross‐kingdom association patterns observed across time points (bacteria–*Candida* correlations) suggest that a time‐dependent ecological shift may emerge during follow‐up, rather than a purely discrete event. Given the limited number of candidiasis cases and the weekly sampling design, the timing of this potential imbalance can only be localized to the interval between two sampling points (e.g., T1–T2 or T2–T3) and cannot be determined more precisely.

Despite this apparent stability, relevant ecological patterns emerged when patients who developed oral candidiasis were compared to those who did not. Candidiasis occurred in 25% of subjects, aligning with previously reported frequencies in corticosteroid‐treated OLP cohorts (Lodi et al. 2007). In these patients, *Candida* proliferation appeared associated with specific bacterial signatures: taxa such as *Streptococcus*, *Rothia*, and *Actinomyces* were consistently more abundant in patients who remained free of fungal infection, whereas anaerobic and proteolytic genera—including *Porphyromonas*, *Prevotella*, and *Lachnoanaerobaculum—showed* positive correlations with *Candida* abundance and were more represented in subjects who developed candidiasis.

The relative enrichment of *Streptococcus* spp. in non‐candidiasis patients may reflect the protective role of these early colonizers in maintaining mucosal homeostasis. *Streptococcus* species are known to produce hydrogen peroxide and bacteriocins, contribute to oxygen stabilization, and create microenvironments that inhibit fungal overgrowth (Bertolini et al. 2021). In contrast, *Porphyromonas* spp., which were positively correlated with *Candida* proliferation in our dataset, are recognized as keystone pathogens in oral dysbiosis. These anaerobic species possess proteolytic and heme‐dependent metabolic capacities that enable them to thrive in oxygen‐depleted niches (Lamont et al. 2018). The concurrent increase of *Porphyromonas* and *Candida* observed in our patients supports a cross‐kingdom interaction model, in which fungal overgrowth consumes oxygen and fosters anaerobiosis, thereby favoring the expansion of pathobionts such as *Porphyromonas* and *Treponema* (Pignatelli et al. 2024).

Interestingly, no specific baseline microbial configuration appeared predictive of subsequent *Candida* overgrowth. This suggests that the onset of oral candidiasis is not determined by an inherently dysbiotic starting point but rather by subtle ecological shifts within the microbial network, possibly triggered or amplified by local immunosuppression during corticosteroid exposure. From a clinical perspective, this reinforces the importance of monitoring to identify early microbial indicators of imbalance before overt infection develops.

This preliminary study has some limitations, primarily related to the small sample size, which reduced the possibility of performing detailed statistical analyses and assessing associations between microbial composition and clinical parameters. Fungal sequencing was feasible only in ten subjects, limiting the statistical power of cross‐domain analyses. Moreover, the lack of quantitative culture data for *Candida* spp. prevents firm conclusions regarding fungal load or strain‐specific behavior. Nevertheless, investigation may support a model in which bacterial–fungal interactions, rather than bacterial dysbiosis alone, underlie the microbial response to corticosteroid therapy in OLP. A preserved abundance of commensal *Streptococcus* and *Rothia* might exert a stabilizing effect against fungal proliferation, whereas an increase in anaerobic taxa such as *Porphyromonas* and *Prevotella* may represent an ecological shift toward conditions that favor *Candida* overgrowth. Understanding these cross‐kingdom interactions will be essential to develop microbiota‐informed management strategies for OLP, including the identification of microbial biomarkers potentially predictive of complications and the possible use of probiotic or microbiota‐modulating adjuncts to reduce secondary infections.

## Author Contributions

**A. Dormiente:** investigation, writing – original draft, methodology, data curation. **L. Mancabelli:** investigation, writing – original draft, methodology, writing – review and editing, software, formal analysis. **M. Ventura:** resources, supervision, writing – review and editing. **G. Longhi:** formal analysis, data curation, writing – review and editing. **G. Mergoni:** funding acquisition, writing – review and editing, validation. **M. Manfredi:** conceptualization, writing – review and editing, methodology, validation, supervision.

## Funding

This study was supported by the University of Parma through the “Bando di Ateneo per la Ricerca 2023‐ Azione B” (Decreto Rettorale DR No. 438, 16/02/2024).

## Ethics Statement

This study was approved by the AVEN (Area Vasta Emilia Nord) Ethical Committee of the University Hospital of Parma, Italy (Study No. 703/2021/TESS/AOUPR). All participants provided written informed consent.

## Conflicts of Interest

The authors declare no conflicts of interest.

## Supporting information


**Table S1:** Summary of sequencing output and read filtering across all samples.
**Table S2:** Bacterial taxonomic profiles of the samples analyzed.
**Table S3:** Taxonomic profiles of Candida species identified by ITS profiling.

## Data Availability

Raw shotgun metagenomic and fungal ITS sequencing data are available in the NCBI Sequence Read Archive (SRA) under accession number PRJNA1391436.
